# NOS3 Polymorphisms and Chronic Kidney Disease

**DOI:** 10.1590/2175-8239-JBN-3824

**Published:** 2018-05-28

**Authors:** Alejandro Marín Medina, Eduardo Esteban Zubero, Moisés Alejandro Alatorre Jiménez, Sara Anabel Alonso Barragan, Carlos Arturo López García, José Juan Gómez Ramos, Juan Francisco Santoscoy Gutierrez, Zurisadai González Castillo

**Affiliations:** 1 Universidad de Guadalajara, Centro Universitario de Ciencias de la Salud, Departamento de Genética, Guadalajara, México; 2 Universidad de Zaragoza, Departamento de Farmacología y Fisiología, Zaragoza, España.; 3 Asociación Mexicana de Atrofia Muscular Espinal, Guadalajara, México.; 4 Universidad de Guadalajara, Centro Universitario de Ciencias de la Salud, Departamento de Neurociencias, Guadalajara, México.; 5 Centro de Investigación Biomédica de Occidente, Guadalajara, México.; 6 University of Texas Health Science Center at San Antonio, Department of Cellular and Structural Biology, San Antonio, United States.; 7 Instituto Mexicano del Seguro Social (IMSS), Hospital General Regional No. 89, Guadalajara, México.; 8 Instituto Mexicano del Seguro Social, Centro de Investigación Biomedica de Occidente, Departamento de Neurociencia, Guadalajara, México; 9 Asociación Mexicana de Atrofa Muscular Espinal (AMAME), Guadalajara, México.

**Keywords:** Renal Insufficiency, Chronic, Nitric Oxide Synthase, Polymorphism, Genetic, Insuficiência Renal, Crônica, Sintase do Óxido Nítrico, Polimorfismo, Genética

## Abstract

Chronic kidney disease (CKD) is a multifactorial pathophysiologic irreversible process that often leads to a terminal state in which the patient requires renal replacement therapy. Most cases of CKD are due to chronic-degenerative diseases and endothelial dysfunction is one of the factors that contribute to its pathophysiology. One of the most important mechanisms for proper functioning of the endothelium is the regulation of the synthesis of nitric oxide. This compound is synthesized by the enzyme nitric oxide synthase, which has 3 isoforms. Polymorphisms in the NOS3 gene have been implicated as factors that alter the homeostasis of this mechanism. The Glu298Asp polymorphisms 4 b/a and -786T>C of the NOS3 gene have been associated with a more rapid deterioration of kidney function in patients with CKD. These polymorphisms have been evaluated in patients with CKD of determined and undetermined etiology and related to a more rapid deterioration of kidney function.

## RESUMO

A insuficiência renal crônica (IRA) é um processo fisiopatológico multifatorial e irreversível que frequentemente conduz a um estado terminal no qual o paciente passa a necessitar de tratamento por transplante renal. A maioria dos casos de IRA são devidos a doenças crônicas degenerativas; a disfunção endotelial é um dos fatores contribuintes na fisiopatologia. Um dos mecanismos mais importantes para o funcionamento adequado do endotélio é a regulação da síntese de óxido nítrico. Este composto é sintetizado por meio da enzima sintase do óxido nítrico, que tem três isoformas. Os polimorfismos no gene *NOS3* tem sido implicados como fatores que alteram a homeostase desse mecanismo. Os polimorfismos *Glu298Asp* 4 b/a e -786T>C do gene *NOS3* têm sido associados a uma deterioração mais rápida da função renal nos pacientes com IRA. Estes polimorfismos têm sido avaliados em pacientes com IRA de causas determinadas ou não-determinadas e relacionados a uma perda mais rápida da função renal.

## INTRODUCTION

In 2002, the *National Kidney Foundation* K/DOQI guidelines defined chronic kidney disease (CKD) as a kidney damage with a duration of three months or longer. This damage can present with structural or functional kidney alterations, with or without decreased glomerular filtration. The structural modifications can be evidenced histologically, radiologically or by biochemical markers of kidney damage in serum or urine samples. The reduced kidney function is manifested by a glomerular filtration rate lower than 60 mL/min/1.73 m^2^ with or without kidney damage (K/DOQI).^1^

The prevalence of chronic kidney disease has been increasing worldwide. In the United States, the prevalence has increased 10% from 1988 to 1994 and 13.1% from 1999 through 2004. In Taiwan, there was an increase of 2% in 1996 and 9.3% in 2003. In a study performed in Japan, an increase of prevalence was observed in males (13.8% in 1974 and 22.1% in 2002), without a significant increase in women.^2^

In Mexico, as in most countries, a marked increase in the prevalence and incidence of CKD has been observed. According to the latest statistics provided by the Instituto Mexicano del Seguro Social (IMSS), it is estimated an incidence of 377 cases per million inhabitants, with a prevalence of 1,142 inhabitants. At present, there are around 52,000 patients undergoing replacement therapy and about 80% of these patients depend on the IMSS. There has been an increase of 92 patients per million inhabitants in 1999 and 400 patients per million inhabitants in 2008.^3^

One of the factors that regulate vascular tone and influence endothelial dysfunction is nitric oxide. This compound is synthesized in the vascular endothelium by the action of the enzyme nitric oxide synthase (NOS).^4^

### NITRIC OXIDE SYNTHASE

The enzyme NOS has 3 isoforms:

- nNOS or NOS type I (neuronal nitric oxide synthase)
- iNOS or NOS type II (inducible nitric oxide synthase)
- eNOS or NOS type III (endothelial nitric oxide synthase).^5^

The gene for nNOS is located at 12q24 and the gene for iNOS is located at 17q11.2. The *NOS3 gene* is described in detail in the next section. Each of these enzymes exhibits certain characteristics that are summarized in Table 1.^6^

**Table 1:** Characteristics of the different isoforms of the enzyme NOS.^6^

Isoform	Features
nNOS	150-160 KDa
	Cytosolic and membrane-bound Constitutive Expression Calcium Dependent Low production of NO (nitric oxide)
iNOS	125-135 KDa
	Predominance of cytosolic Inducible Expression Not dependent on calcium High production of ON
eNOS	135 KDa
	Mainly coupled with membrane Constitutive Expression Calcium Dependent Low production of ON and posttranslational myristoylation palmitoylation

The most important isoform for this revision is the endothelial nitric oxide synthase (eNOS).

### NOS3 GENE

Genome-wide association studies (GWAS) offer the possibility of finding candidate genes for susceptibility to kidney disease and the progression of CKD (Staples et al, 2010 Risk Factors for progression of chronic kidney disease). One of these genes is the *NOS3, which has 23*.605 bases and is located at 7q36.1. It has 26 exons and encodes for eNOS , an enzyme composed of 1203 amino acids with a molecular weight of 133 289 Da.^7^^,^ 8

The enzyme acts as a homodimer and is located in the cell membrane, cytoplasm, and Golgi apparatus. The enzyme uses 5 cofactors:

- A heme
- FAD (flavin adenine dinucleotide)
- FMN (flavin mononucleotide)
- BH4 (tetrahydrobiopterin cofactor)
- NADPH (nicotinamide adenine dinucleotide phosphate).^9^

**Figure 1:**
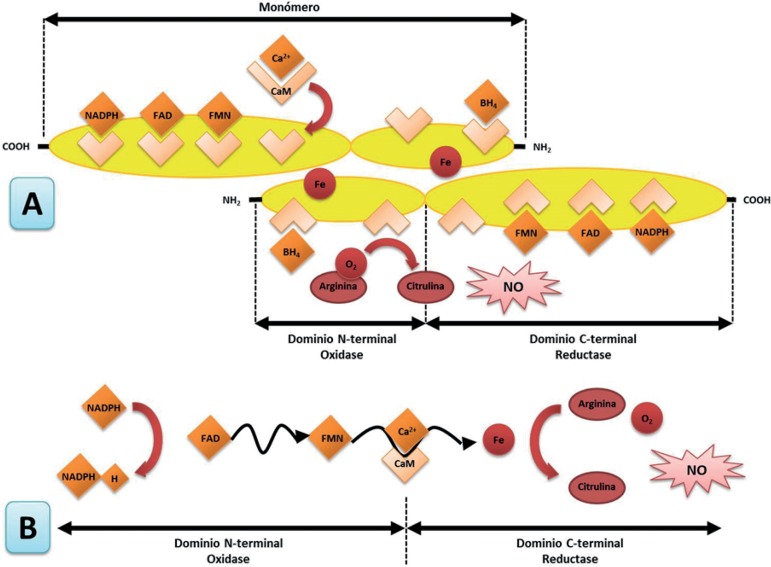
A) Structure of the enzyme endothelial nitric oxide synthase. B) Electron transfer mechanism. Modified from Dias RG, et al.^10^

The enzyme catalyzes the conversion of L-arginine into nitric oxide (Figure 1). The synthesis is performed in 2 reactions. First, the enzyme works as an arginine hydroxylase. In the second reaction, it acts as hydroxyarginine monooxygenase. In this reaction, there is a net transfer of five electrons, four of them necessary to reduce O_2_ from the NADPH and from arginine. In the first reaction, the NADPH assigns two electrons, which oxidize the nitrogen of the guanidine group of arginine. In the second step, the NADPH provides an electron and the N-hydroxyarginine experiences an oxidation of three electrons to form citrulline and nitric oxide.^10^^,^ 11

In the amino-terminal end, the enzyme has an oxygenase domain, which contains the catalytic site and the binding sites for BH4, heme, and L-arginine (Figure 1). In the carboxyl terminal, the enzyme has a domain of reductase, which contains binding sites for NADPH, FMN, and FAD; the two domains are linked in its central part by a domain that secures the calmodulin.^12^^,^ 13

The activity of the enzyme is regulated by free calcium and the subsequent union of the calcium-calmodulin complex. Hormonal factors such as pregnancy and increased estrogen levels enhance the expression of the enzyme.^6^

The final product from the enzyme reaction is nitric oxide (NO), which is a gas that easily disseminates from the endothelial cells to the smooth muscle cells of the vascular wall. NO has very short half-life of 0.5 to 5 seconds and quickly metabolizes to nitrites, which can be measured indirectly.^14^

### NITRIC OXIDE AND KIDNEY DISEASE

Renal nitric oxide plays several hemodynamic functions in the renal glomeruli. However, its most important effect is the promotion of diuresis and natriuresis, as well as renin secretion regulation. The eNOS is expressed in large amounts in the renal vascular endothelium (including the afferent and efferent arterioles). It is also expressed in the proximal tubule, the thick portion of the ascending loop of Henle, and the collecting tubule. The precise role of nitric oxide in the proximal tubules is unknown; however, in a study conducted in mice where the expression of this enzyme was abolished, an increased reabsorption of NaCl was observed, which caused an increased glomerular filtration rate (GFR) favoring the emergence of hypertension.^15^

In kidney disease, the production of nitric oxide reduces either by a decrease in the enzyme substrate (L-arginine), or by an increase in the bioavailability of the enzyme inhibitor asymmetric dimethylarginine (ADMA), which in turn decreases the synthesis of nitric oxide by a feedback mechanism. This mechanism has been found to accelerate in the progression of a pre-existing kidney disease.^16^

### POLYMORPHISMS IN THE GENE NOS3

A polymorphism is defined as a genetic variant that is present in more than 1% of the population. Three main polymorphisms of *NOS3* gene have been studied in different diseases found to be associated with diabetic nephropathy in different studies:^16^^,^ 17 894G>T or Glu298Asp (rs1799983), 27-bp repeat in intron 4 (VNTR) 4b/a variants, and -786 T>C.

### 894G>T OR GLU298ASP POLYMORPHISM (RS1799983)

The 894G>T polymorphism, localized in exon 7, consists of a change of glutamic acid to aspartic acid (Glu298Asp).^18^ The change of glutamate (E) by aspartic acid (D) affects the domain of the oxidase enzyme, which is the binding site for BH4 and the amino acid L-arginine. The change causes an enzyme variation, making it more susceptible to proteolytic cleavage in position D238-P239. It generates a shorter form of the enzyme and therefore causes less production of NO.^9^

This polymorphism has been primarily associated with different cardiovascular diseases, such as coronary artery disease, atherosclerosis, coronary spasm induced by acetylcholine, and arterial hypertension. Other diseases related with this polymorphism are Alzheimer's disease, pregnancy-induced hypertension, bladder cancer, prostate cancer, diabetic nephropathy, among many others.^17^

Studies in African, Caucasian, and African-American populations have found that the frequency of the polymorphic allele (T) in the general population is 14.3%, 40.4%, and 66.1% respectively.^19^ In the Mexican mestizo population, Rosas-Vargas- et al.,^20^ in a study with 126 patients, reported a frequency of 23% for this polymorphism.

Different studies have reported an association between this polymorphism and chronic kidney disease.^21^ In a study carried out in 37 Mexican patients diagnosed with the renal variant of Fabry disease, the authors found an association between Asp298 and 4a alleles of the *NOS3* gene. It was noted that patients with these alleles had an increased level of urea and creatinine, and a decrease in glomerular filtration rate. The association behaved under a co-dominant inheritance model.^22^

In other studies, the presence of the T allele (aspartic acid) has been associated with a susceptibility to CKD development in several populations, especially in the Asian population.^23^ This was observed in another study carried out in India with CKD secondary to diabetic nephropathy, where an increase in creatinine levels was found in patients with the asp298 allele. Also, a statistically significant association with oxidative stress markers such as *SOD2* and *GST* was observed.^24^

However, in another study conducted in Malaysia, there was no association between these genetic markers and CKD.^25^

### *4 B/A* (VNTR IN INTRON 4)

This polymorphism is composed of 27 pairs of bases, characterized by allele "a" that contains 4 repeated (deletion/polymorphic) modifications and allele "b" that consists of 5 (push/wild) modifications. Recent studies suggest that this polymorphism may regulate the expression of this gene through the production of small interfering RNA (iRNA) of 27 nucleotides, decreasing the expression of the gene and/or synthesis of the protein.^26^

In studies carried out in African, Caucasian, and African-American populations, the frequency of this polymorphism in the general population is 36%, 29.7%, and 36.1%, respectively.^27^

In a Brazilian study on CKD, a significant increase in the frequency of allele "a" in these patients was found compared to the controls, and a strong statistical association was noticed between this allele and the disease.^28^

However, in another study carried out in Sweden and Finland in a population with chronic renal disease secondary to diabetic nephropathy, a low frequency of the allele 4a was observed and no statistical association was seen between the polymorphism and the disease.^29^

### *-786 T>C* (RS2070744)

This polymorphism, situated in the flanking region 5', has been associated with a decrease in the expression of the *NOS3* gene, as it decreases the transcription rate of the gene by 50%. It is thought that it can bind to the replication protein A1; this protein participates in several cellular processes, among them transcription.^30^^,^ 31

In studies conducted in African, Caucasian, and African-American populations, the frequency of this polymorphism is 3.2%, 14.5%, and 1.8%, respectively.^26^

In a study carried out in an Indian population with CKD, a high frequency of the asp298, -T786C, and 4a alleles polymorphisms was found and their nitrite levels were lower in comparison with the controls. Therefore, it was determined that there is an association between these polymorphisms and chronic kidney disease.^32^

However, in another study performed in a Brazilian Caucasian population, there was no association between these polymorphisms and CKD.^33^

## CONCLUSIONS

The functional integrity of the endothelium allows for a precise balance between vasoconstrictor and vasodilator agents. In normal conditions, there is a predominance of vasodilator, anticoagulant, and antiproliferative (mainly NO) agents over the vasoconstrictor, procoagulant, and proliferative agents. The polymorphisms already mentioned in the *NOS3 gene* have been associated with endothelial dysfunction in different populations. However, some studies did not find an association; therefore, the results are controversial. Further studies should be conducted in different populations to identify potential genetic risk factors for CKD.
